# Tracheal Mucoepidermoid Carcinoma Mimicking Deteriorated Bronchial Asthma during Pregnancy

**DOI:** 10.1155/2021/7259496

**Published:** 2021-06-29

**Authors:** Takeshi Murakami, Yasuyuki Fujita, Kazuhiro Takamura, Shuichi Taniguchi, Chikara Fukuyama, Kousuke Marutsuka, Tomihiro Shimamoto

**Affiliations:** ^1^Department of Obstetrics and Gynecology, Miyazaki Prefectural Miyazaki Hospital, Japan; ^2^Department of Respiratory Medicine, Miyazaki Prefectural Miyazaki Hospital, Japan; ^3^Department of Pathology, Miyazaki Prefectural Miyazaki Hospital, Japan

## Abstract

Primary bronchial tumors are extremely rare. However, symptoms, such as coughing and wheezing, are not specific to this disease, and primary bronchial tumors are often misdiagnosed as bronchial asthma. This report describes the case of a pregnant patient with a bronchial tumor that mimicked deteriorating bronchial asthma. A 37-year-old female patient suffered from repeated episodes of pneumonia since 26 weeks of gestation. Despite treatment, she suffered from another episode of pneumonia at 28 weeks of gestation. This was considered as deteriorating asthma. Bronchoscopy performed at 34 weeks of gestation showed a tumor in the left main lung bronchus, obstructing nearly 100% of the trachea. After cesarean delivery at 34 weeks, she underwent endoscopic bronchial tumor resection. Because of recurrent bronchial obstruction and the possibility of malignant disease, subsequent left main lung bronchial resection and bronchoplasty were performed. The pathological diagnosis was low-grade mucoepidermoid carcinoma. In conclusion, if pneumonia develops repeatedly during pregnancy, the possibility of bronchial tumor should be considered.

## 1. Introduction

Primary bronchial tumors are extremely rare [1, 2]. As the disease progresses, the tumor obstructs the airway, resulting in symptoms such as coughing, wheezing, pneumonia, and dyspnea. Since these symptoms are not specific to this disease, primary bronchial tumors are often misdiagnosed as bronchial asthma or chronic obstructive pulmonary disease [2]. This case report describes a bronchial tumor which mimicked deteriorating bronchial asthma in a pregnant patient.

## 2. Case Presentation

**Figure 1:**
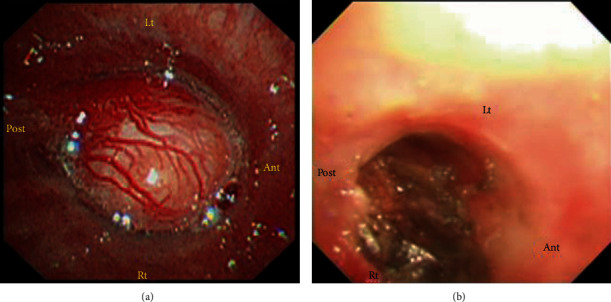
Bronchoscopic images taken at 34 weeks showed a bronchial tumor of 15 mm in the left main bronchus (a). This lesion was removed after delivery using bronchoscopic surgery (b). Lt: left; Ant: anterior; Rt: right; Post: posterior.

**Figure 2:**
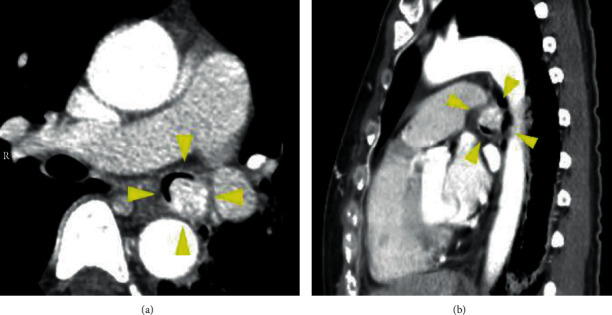
Transverse (a) and sagittal view (b) of a chest CT, taken after delivery, revealed a bronchial tumor in the left main bronchus without invasion to surrounding organs (yellow arrowheads).

**Figure 3:**
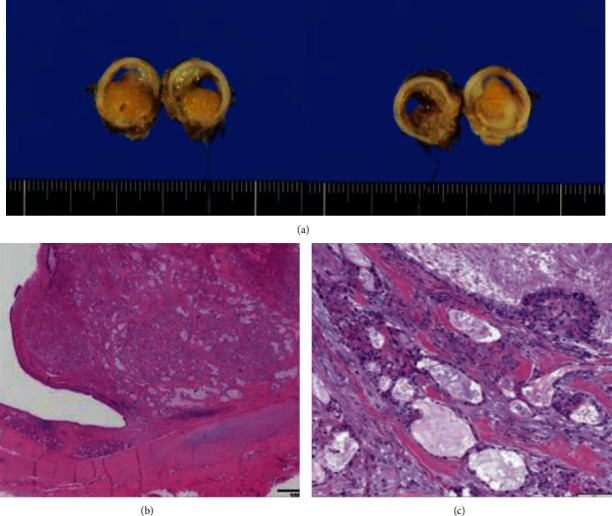
Macroscopic and microscopic findings of the resected bronchial tumor. The excised specimen occupying the bronchi was soft (a). Pathologically, a nodular polypoid tumor is arising from the bronchial wall, based mainly at the membranous portion consisting of a mixture of epithelial and fibromyxoid stromal elements. In addition, neither a high mitotic activity nor extrabronchial invasive growth is noted, indicating the diagnosis of a low-grade mucoepidermoid carcinoma (b, c).

The patient was a 37-year-old woman, G2P1, with an unremarkable family history. She had been diagnosed with bronchial asthma by her family doctor at the age of 33 years. The use of steroid inhalation therapy did not appear to impact her condition [3]. Her previous pregnancy had resulted in an emergency cesarean delivery at 33 weeks of gestation due to severe hypertensive disorders of pregnancy (HDP) and fetal growth restriction (FGR). She became pregnant for the second time and was referred to our hospital owing to her previous birth history. Her estimated due date was confirmed from the CRL value during the first trimester. Her pregnancy was uneventful; however, she developed fever and cough at 26 weeks of gestation and was subsequently diagnosed with sinusitis due to deteriorated bronchial asthma. She was admitted to the hospital under the care of the respiratory team and was administered ceftriaxone. However, despite administration of antibiotics, the wet cough associated with asthma persisted. She suffered from pneumonia at 28 weeks of gestation, and atelectasis was detected in the left lower lobe using a chest radiograph. Although her symptoms began to improve, bronchoscopy was performed at 34 weeks of gestation due to recurrent pneumonia. Bronchoscopy showed a tumor with a smooth surface measuring approximately 1.5 cm in the left main lung bronchus. The tumor obstructed nearly 100% of the trachea (Figure 1(a)) [3]. Further enlargement of the mass would have led to complete tracheal obstruction, suggesting a risk of sudden left lung atelectasis and maternal hypoxia; therefore, early medical intervention was necessary. Additionally, her blood pressure was elevated at 32 weeks of gestation and fetal growth was restricted at -2.0 SD of normal fetal growth, so that she was diagnosed with preeclampsia (PE-EO). Following counseling with the patient, her family, and respiratory medicine doctors, a bronchoscopic tumor resection was planned following the delivery of the baby. A cesarean section was performed at 34 weeks of gestation due to previous cesarean delivery. She delivered a female infant weighing 1322 g, with Apgar score of 8 and 9 points at 1 minute and 9 points at 5 minutes, respectively. The pH of umbilical artery blood gas was 7.321. A chest CT taken after delivery revealed a bronchial tumor in the left main bronchus without invasion to surrounding organs (Figure 2), and the patient underwent rigid endoscopic bronchial tumor resection 7 days after the cesarean section (Figure 1(b)) [3]. The histological type could not be identified at this point, and left lung atelectasis developed due to postoperative inflammatory changes. CT examination 4 weeks after the endoscopic resection confirmed relapse of bronchial tumor. Owing to obstruction by the recurrent bronchial tumor and to rule out the possibility of malignancy, left main lung bronchial resection and bronchoplasty were performed 6 weeks after the endoscopic resection. The pathological diagnosis of the resected specimen was a low-grade mucoepidermoid carcinoma (Figure 3). No postoperative adjuvant treatment was needed, and her postoperative course was uneventful. There was no recurrence evident 18 months later.

## 3. Discussion

Tracheal and bronchial tumors are rare, and of these, mucoepidermoid carcinoma (MEC) is especially unusual, accounting for 0.1 to 0.2% of all primary lung tumors [1]. Since more than 50% of tracheal and bronchial tumors develop in patients less than 30 years old [4], it is quite possible for them to be associated with pregnant women. The symptoms of bronchial tumors as well as MECs depend on the degree of tracheal obstruction; therefore, the size and location of the tumor are important. Symptoms such as coughing, wheezing, and occasional hemoptysis are often misdiagnosed as other bronchial diseases such as bronchial asthma and COPD [1, 4–7]. Obstructive symptoms are said to occur with airway obstruction of 50-75% [7] and may be asymptomatic. Although obstructive pneumonia may occur repeatedly due to airway obstruction, correct diagnosis may be delayed due to other nonspecific symptoms. Further, bronchial tumors often mimic bronchial asthma [1]. In this case, the patient was diagnosed with bronchial asthma at the age of 33 years and underwent steroid therapy. However, the treatment was inadequate. Retrospectively, these symptoms were likely to be manifestations of early stage bronchial tumors.

CT examination is useful for confirming the spread of the tumor and its relationship with various tissue types [1, 5]. In addition, calcification, which is specific to MECs, may be observed in tracheobronchial tumors [1, 5]. This information is important when deciding on a surgical treatment strategy. On a chest radiograph, pneumonia and atelectasis may be apparent, as in our case; however, specific findings which would point towards a bronchial tumor maybe not be identified on a radiograph [1, 5].

MECs, which were diagnosed in this case, are classified into high grade and low grade based on their histology. High-grade tumors have a poor prognosis, whereas low-grade tumors grow slowly and are more likely to represent benign disease. A previous report discusses a case in which laser treatment was used to manage bronchial tumors during pregnancy [6]. The authors emphasized that close attention should be paid towards maternal hypoxia and fetal well-being when using laser treatment in pregnant patients. In other case reports, the tumor was resected during or after delivery, as in our case. In cases where a low-grade tumor is diagnosed histopathologically, treatment should be withheld until fetal maturity. On the other hand, in cases of high-grade tumors and tumors where the grade cannot be identified, immediate treatment is preferred. In addition, the size of tumor and the clinical manifestations also determine the timing of delivery in our case.

Chronic hypoxia caused by respiratory diseases such as bronchial asthma and pulmonary tuberculosis may increase the incidence of pregnancy-hypertension syndrome and subsequently result in fetal growth restriction [8]. In this case, the patient had developed preeclampsia during pregnancy three years previously. In this pregnancy, the patient suffered preeclampsia with fetal growth restriction (FGR). The MEC in this case was of a low grade; it may have existed for over 3 years and resulted in bronchial obstruction. Thus, the patient's chronic hypoxia may have been the cause of her preeclampsia and FGR.

In conclusion, although bronchial tumors are rare, they occur more commonly in people of childbearing age. Therefore, if pneumonia occurs repeatedly during pregnancy, the possibility of a bronchial tumor should be kept in mind, and CT examination and/or bronchoscopy should be considered.
